# Polysaccharide Chain Length of Lipopolysaccharides From *Salmonella* Minnesota Is a Determinant of Aggregate Stability, Plasma Residence Time and Proinflammatory Propensity *in vivo*

**DOI:** 10.3389/fmicb.2019.01774

**Published:** 2019-08-02

**Authors:** Wahib Sali, Danish Patoli, Jean-Paul Pais de Barros, Jérôme Labbé, Valérie Deckert, Vincent Duhéron, Naig Le Guern, Denis Blache, Denis Chaumont, Eric Lesniewska, Benoit Gasquet, Catherine Paul, Mathieu Moreau, Franck Denat, David Masson, Laurent Lagrost, Thomas Gautier

**Affiliations:** ^1^LipSTIC LabEx, UMR1231, Lipids Nutrition Cancer, Inserm/University of Bourgogne Franche-Comté, Dijon, France; ^2^UMR6303 Laboratoire Interdisciplinaire Carnot de Bourgogne, CNRS/University of Bourgogne Franche-Comté, Dijon, France; ^3^Cell Imaging platform, Inserm/University of Bourgogne Franche-Comté, Dijon, France; ^4^Laboratoire d’Immunologie et Immunothérapie des Cancers, EPHE, PSL Research University, Paris, France; ^5^LIIC, EA7269, University of Bourgogne Franche-Comté, Dijon, France; ^6^Institut de Chimie Moléculaire de Bourgogne, UMR6302, CNRS/University of Bourgogne Franche-Comté, Dijon, France; ^7^University Hospital of Dijon, Dijon, France

**Keywords:** aggregation, inflammation, lipopolysaccharides, mouse, pharmacokinetics

## Abstract

Lipopolysaccharides (LPS) originate from the outer membrane of Gram-negative bacteria and trigger an inflammatory response *via* the innate immune system. LPS consist of a lipid A moiety directly responsible for the stimulation of the proinflammatory cascade and a polysaccharide chain of variable length. LPS form aggregates of variable size and structure in aqueous media, and the aggregation/disaggregation propensity of LPS is known as a key determinant of their biological activity. The aim of the present study was to determine to which extent the length of the polysaccharide chain can affect the nature of LPS structures, their pharmacokinetics, and eventually their proinflammatory properties *in vivo*. LPS variants of *Salmonella* Minnesota with identical lipid A but with different polysaccharide moieties were used. The physical properties of LPS aggregates were analyzed by zetametry, dynamic light scattering, and microscopy. The stability of LPS aggregates was tested in the presence of plasma, whole blood, and cultured cell lines. LPS pharmacokinetics was performed in wild-type mice. The accumulation in plasma of rough LPS (R-LPS) with a short polysaccharidic chain was lower, and its hepatic uptake was faster as compared to smooth LPS (S-LPS) with a long polysaccharidic chain. The inflammatory response was weaker with R-LPS than with S-LPS. As compared to S-LPS, R-LPS formed larger aggregates, with a higher hydrophobicity index, a more negative zeta potential, and a higher critical aggregation concentration. The lower stability of R-LPS aggregates could be illustrated *in vitro* by a higher extent of association of LPS to plasma lipoproteins, faster binding to blood cells, and increased uptake by macrophages and hepatocytes, compared to S-LPS. Our data indicate that a long polysaccharide chain is associated with the formation of more stable aggregates with extended residence time in plasma and higher inflammatory potential. These results show that polysaccharide chain length, and overall aggregability of LPS might be helpful to predict the proinflammatory effect that can be expected in experimental settings using LPS preparations. In addition, better knowledge and control of LPS aggregation and disaggregation might lead to new strategies to enhance LPS detoxification in septic patients.

## Introduction

Lipopolysaccharides (LPS, endotoxins) are amphipathic molecules that originate from the outer membrane of Gram-negative bacteria. They trigger the first step of the innate immune response by interacting with the CD14/TLR4/MD2 receptor complex at the surface of leukocytes (Beutler and Rietschel, 2003). The inflammatory response resulting from this initial stimulus consists of the production of proinflammatory cytokines. If uncontrolled, the response may lead to septic shock, which is characterized by hemodynamic dysfunctions, multiple organ failure, and death of the host organism (Van Amersfoort et al., 2003). Better understanding of the parameters that influence LPS toxicity, inactivation, and clearance is crucial to set up new strategies aimed at controlling the inflammatory burst in septic patients. In addition, it may help to predict the proinflammatory propensity of LPS preparations in experimental settings.

LPS are complex glycolipids containing an O-antigen polysaccharide, a core oligosaccharide, and an amphipathic lipid A moiety (Raetz and Whitfield, 2002). The O antigen consists of the variable repetition of an oligosaccharide unit. Due to its high variability in terms of length and the nature of repeats, the O antigen determines the antigenic specificities between and within bacterial strains (Raetz and Whitfield, 2002). Lipid A usually contains six or seven fatty acid residues linked to two phosphorylated glucosamine sugars. As an example, LPS from *Salmonella enterica* subsp. *enterica serovar* Minnesota was described to harbor, from the hydrophilic to the hydrophobic end: (1) an O-antigen consisting mostly of 1–2 (for 55%) or 20 repeating units (41%); (2) a core containing 5 hexose, 3 heptose, and 3 Kdo (2-keto-3-deoxyoctonate) residues; and (3) a lipid A moiety with a diphosphorylated diglucosamine backbone carrying 7 fatty acids (including four 3-hydroxymyristate residues) that are distributed according to a 5 + 2 asymmetric pattern (Peterson and McGroarty, 1985; Kanegasaki et al., 1986; Brandenburg et al., 1999). The lipid A moiety represents the active, proinflammatory moiety of LPS molecules as it binds directly to TLR4 and MD2 (Park et al., 2009). Consequently, variations in lipid A structure are known to alter the proinflammatory properties of LPS (Wilkinson, 1996) and have been the subject of intense research and attention, especially regarding fatty acid composition and phosphorylation (Qureshi et al., 1982; Brandenburg et al., 2000; Seydel et al., 2003; Tanimura et al., 2014).

Besides the lipid A structure, the length of the polysaccharide chain could partly explain the variability of the endotoxic activity of LPS (Vukajlovich and Morrison, 1983; Kitchens and Munford, 1998; Mueller et al., 2004, 2005; Hardy et al., 2012). Indeed, this hydrophilic moiety of LPS has an impact on LPS solubility in the aqueous phase (Wilkinson, 1996), and because they are amphipathic compounds, purified LPS are rarely found as monomers but rather in the form of aggregates in aqueous media (Brandenburg et al., 2003; Sasaki and White, 2008). Moreover, it should be stressed that most LPS in nature are embedded in the outer membrane of multiplying or dying bacteria or in LPS-rich blebs (Brandenburg et al., 2003). Consequently, the nature and structural properties of LPS aggregates might influence the accessibility of LPS molecules to CD14, MD2, and TLR4 receptors (Tobias et al., 1995; Gioannini et al., 2004; Mueller et al., 2005; Park et al., 2009) as well as their behavior in the circulation (Gautier et al., 2008, 2010).

Although the length of the polysaccharide chain might influence the occurrence, size, and structure of LPS aggregates (Aurell and Wistrom, 1998; D’Errico et al., 2010; Richter et al., 2011), the biological consequences are largely unknown. Because earlier studies compared LPS from different bacterial strains, which differed in both the lipid A and polysaccharide moieties, it was not possible to work out the real contribution of the hydrophilic chain to the endotoxic potential of the entire LPS molecule (Raetz and Whitfield, 2002; Brandenburg et al., 2003). Interestingly, Hardy and Colleagues reported that reconstituted aggregates containing LPS with a long polysaccharide chain tended to be better able than LPS with a short polysaccharide chain to activate TLR receptors (Raetz and Whitfield, 2002). However, this earlier work was restricted to *in vitro* studies of the ability of the different aggregates to activate signal transduction in a transgenic cell model expressing TLR4 and MD2 together with a reporter gene. To our knowledge, the influence of the length of the polysaccharide chain on the kinetics as well as on the proinflammatory potency of LPS has not yet been studied *in vivo*.

Earlier studies reported that LPS disaggregation, transport, and detoxification as mediated by circulating plasma factors can influence its bioavailability and inflammatory properties (Munford et al., 1981; Schumann et al., 1990; Gautier and Lagrost, 2011). The main goal of the present study was to investigate whether the length of the polysaccharide chain could also modulate the pharmacokinetics and the biological properties of LPS. Previous reports showed indeed that LPS clearance rates can markedly influence the inflammatory and immune response *in vivo* (Harris et al., 1993; Cai et al., 2008; Lu and Munford, 2011). Importantly, previous reports showed that LPS from different bacterial strains or serotypes showed different time courses of plasma clearance or organ uptake (Mathison and Ulevitch, 1979; Freudenberg et al., 1980, 1982). However, no specific attention was paid to the contribution of polysaccharide chain length to the plasma distribution and inflammatory propensity of LPS. In the present work, we compared the physicochemical, pharmacokinetic, and pro-inflammatory properties of two LPS molecules with identical active lipid A moieties but with polysaccharide chains of various lengths *in vivo*. The LPS molecules came from two *Salmonella* Minnesota strains, one of which has a short polysaccharide chain (rough LPS, i.e., leading to the formation of colonies with a rough aspect) and the other one a long polysaccharide chain [smooth LPS, i.e., associated with colonies with a smooth aspect (Raetz, 1990)]. Smooth and rough LPS were injected into wild-type mice to determine the time course of cytokine production, LPS clearance from plasma, and organ uptake. In parallel, dynamic light scattering (DLS), zetametry, steady-state fluorescence analysis of pyrene probes, and transmission electron and atomic force microscopy were combined to explore the physicochemical nature of LPS aggregates.

## Materials and Methods

### Reagents and materials

**Figure 1:**
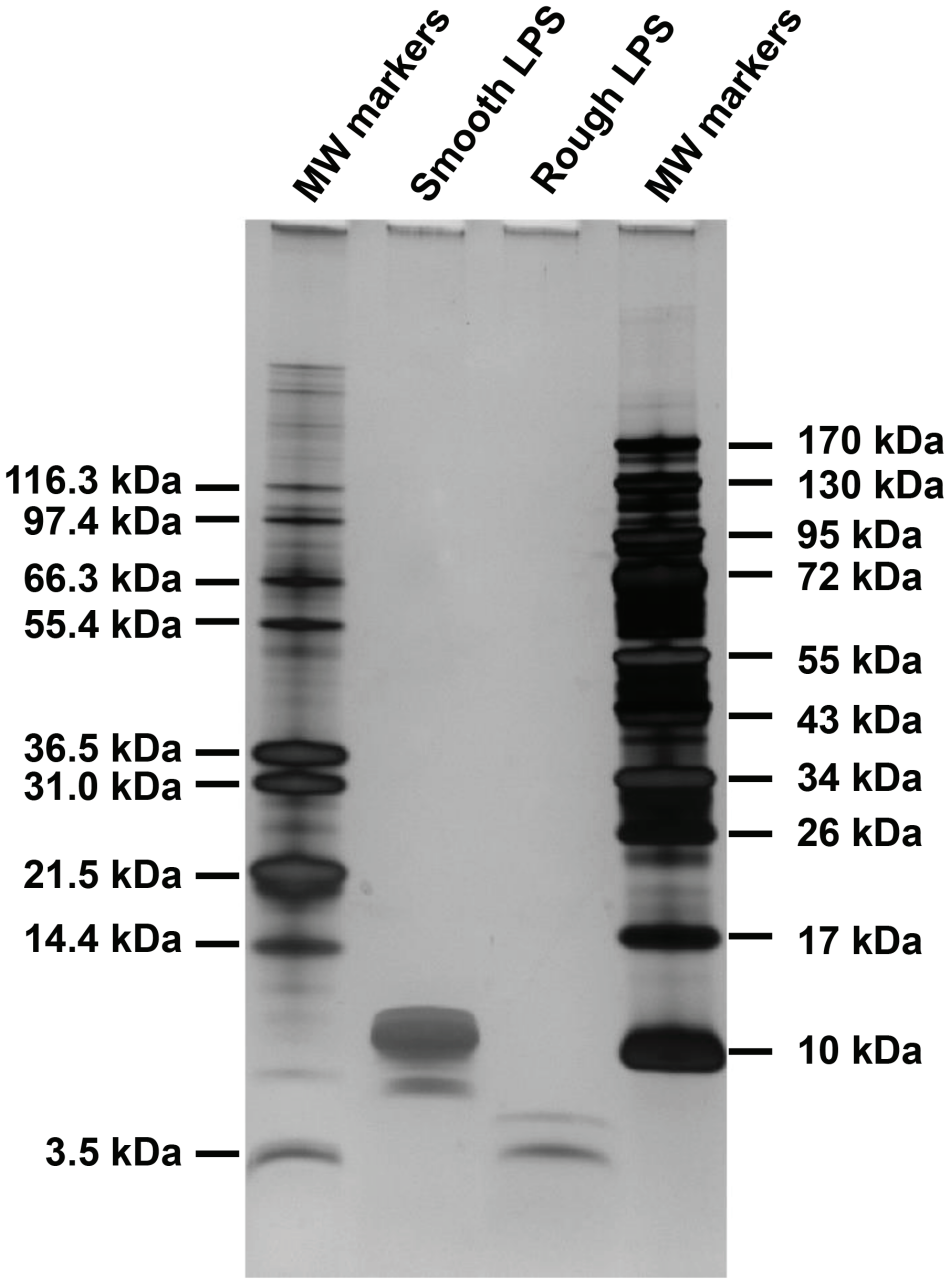
Difference in size between smooth and rough LPS molecules. Briefly, 0.8 μg of smooth and rough LPS were submitted to electrophoresis on a 10–20% polyacrylamide gradient gel in the presence of SDS and detected by silver staining. Apparent molecular weights of major bands, determined by comparison with molecular weight standards that were submitted to electrophoresis together with the samples, were 10.2 kDa for smooth LPS and 3.5 kDa for rough LPS.

LPS purified from *Salmonella enterica* subsp. *enterica serovar* Minnesota S strain (smooth LPS) and Re595 mutant strain (rough LPS) were purchased from Sigma-Aldrich (Saint Louis, MO, USA). The molecular weight of our LPS molecules was checked by polyacrylamide gel electrophoresis. Briefly, 0.8 μg of LPS was heated at 70°C for 10 min in a Tricine buffer containing SDS (Thermo Fisher, Illkirch, France) and submitted to electrophoresis on a 10–20% polyacrylamide gradient gel in Tricine SDS buffer (Novex, Thermo Fisher) prior to silver staining (Pierce Silver Staining Kit, Thermo Fisher). Apparent molecular weights were determined by comparison with protein standards (Mark 12, Thermo Fisher; Protein Ladder, Euromedex, Souffelweyersheim, France) that were submitted to electrophoresis together with the samples, and calculations were performed by using GraphPad Prism 6 software with interpolation from nonlinear regression curve. Smooth LPS showed one major band with a calculated molecular weight of 10.2 kDa and one minor band of 8.7 kDa, while rough LPS displayed a major band of 3.5 kDa and a minor band of 4.5 kDa (Figure 1). For all experiments, lyophilized LPS were dissolved in endotoxin-free saline (0.15 mol/L sodium chloride; B Braun Medical, Melsungen, Germany) and vigorously mixed for 15 min before use. For each LPS preparation, an aliquot was analyzed for molar titration by liquid chromatography-tandem mass spectroscopy (LCMS^2^, see below). All materials were of pyrogen-free grade or made apyrogenic by overnight heating at 150°C, and all the reagents used were of “endotoxin-free” grade. As assessed by LCMS^2^, only trace amounts of LPS were detected in experimental media that were not supplemented with exogenous LPS.

### Animals and Ethics Statement

Wild-type, C57BL/6 J littermate mouse females were purchased from Charles River. The mice were fed a standard chow diet (A03 diet; SAFE, Augy, France) and had free access to food and water. All experiments were performed in accordance with institutional guidelines and approved by the Ethics Committee of the University of Burgundy (Protocol number 2511).

### Lipopolysaccharides Injection and Sampling

LPS preparations were injected intravenously *via* the tail vein (200 μl at 20 μmol/L; single dose) and blood was collected at the indicated times by retro-orbital puncture into endotoxin-free, heparin-containing tubes. After 24 h, the animals were killed by cardiac puncture, and the livers were snap-frozen in liquid nitrogen. Plasma was obtained from blood samples (10 min, 2,000 × *g* at 4°C). All samples were stored at −80°C until further analysis.

### Cytokine Measurements

Interleukin (IL) 6, IL-10, tumor necrosis factor alpha (TNF-α), macrophage chemoattractant protein 1 (MCP-1), and interferon gamma (IFN-γ) in mouse plasma were assayed by cytometric bead array using commercially available kits (CBA mouse inflammation kit, BD Biosciences, San Diego, CA, USA) according to the manufacturer’s instructions. Signal quantification was performed on a Guava EasyCyte Plus flow cytometer (Millipore, Billerica, MA, USA) and analyzed with FCAP Array software (SoftFlow, Pecs, Hungary).

### Lipopolysaccharides Quantitation

LPS in plasma and organs as well as in preparations of smooth and rough LPS was assayed by using the direct quantitation of 3-hydroxymyristic acid, which is a specific component of the lipid A moiety of LPS, by LCMS^2^ using the general procedure previously described (Pais de Barros et al., 2015). Quantitation was achieved by calculating the ratio between ion peaks corresponding to 3-hydroxymyristic acid, and 3-hydroxytridecanoic acid used as an internal standard.

### Atomic Force Microscopy

LPS droplets were deposited on freshly cleaved mica surfaces at room temperature. Samples were imaged with a Nanoscope V Multimodal 8 apparatus (Bruker AXS, Santa Barbara, CA, USA) with a force modulation setup. Piezoelectric scanners (15 μm range) were used in the contact force mode, with scan rates of 1 Hz in association with silicon nitride (SI3N4) cantilevers (Nanoprobes NPS, Bruker AXS, Santa Barbara, CA). The nominal imaging force during scanning was ~15.8 nN (*k* = 0.06–0.56 N/m). All reported images were made with 512 × 512 pixel definition.

### Transmission Electron Microscopy

LPS solutions were stained with either 3% phosphotungstic acid or ammonium heptamolybdate, washed by centrifugal filtration, and applied onto a collodion-coated microscope cover grid. Dried samples were imaged with a Hitachi H7500 energy filtering transmission electron microscope (Hitachi, Bron, France) operating at 80 kV. Digital images were recorded with an Advantage CCD camera driven with AMT software (AMT Imaging, Danvers, MA, USA). All reported images were made with 1,024 × 1,024 pixel definition.

### Size Distribution and Zeta Potential Measurements

Both parameters were determined by using a Malvern Zetasizer Nano ZS device (Malvern, UK). LPS samples in saline were poured into folded capillaries for analysis at a constant temperature of 25°C. Particle size distribution was determined with the dynamic light scattering mode (DLS) over a 0.6–6,000 nm size range. Briefly, samples were illuminated by a 633-nm helium-neon laser, and the scattered light was measured at a backscatter angle of 173° using an avalanche photodiode. Zeta potential, i.e., electronic charge at the slipping plane surrounding the particles in aqueous solutions was measured with the electrophoretic light scattering mode (zetametry) by determining particle mobility in solutions when subjected to an electric field (70 mV). Raw DLS and zetametry data were analyzed and interpreted with Malvern software.

### Steady-State Fluorescence of Pyrene Probes

The hydrophobicity of LPS aggregates was determined by using the pyrene fluorescence peak I to peak III ratio method. Briefly, an apyrogenic phosphate-buffered saline saturated with the pyrene probe (Sigma-Aldrich) was filtered and added to LPS solution in saline (final LPS concentration, 20 μmol/L), and fluorescence was measured at room temperature on an LS50B spectrofluorometer (Perkin Elmer, Waltham, MA, USA). Pyrene emission fluorescence spectra were scanned from 350 to 400 nm with an excitation wavelength of 335 nm. The hydrophobic ratio was calculated by dividing the intensity of the first fluorescence peak (peak I–374 nm) by that of the third peak (peak III–384 nm). Hydrophobicity correlates inversely with the I–III ratio.

### Lipopolysaccharides Fluorescent Labeling

Labeling of smooth and rough LPS with DOTA-Bodipy was performed essentially according to the technique described earlier (Duheron et al., 2014). LPS molecules were resuspended at a concentration of 0.5 mM in sodium bicarbonate buffer pH 8.77. One equivalent of DOTA-Bodipy-NCS that was prepared as previously described (Bernhard et al., 2010) was added prior to incubation for 1 h at 37°C. Labeled LPS was separated from free label by FPLC using a Superdex75 column (GE Healthcare), and fractions containing DOTA-Bodipy-LPS were concentrated in sterile PBS with Nanosep MWCO 3 kDa (Pall).

### Lipopolysaccharides Incubation in Plasma

LPS labeled with DOTA-Bodipy (20 μM) were incubated in plasma from wild-type mice or in PBS in a final volume of 200 μl. Incubations were performed in the dark for 20 min at 37°C with mild agitation and were stopped by transferring reaction tubes on ice. Samples were subsequently submitted to size exclusion chromatography with a Superose 6 HR 10/30 column (GE Healthcare) at a flow rate of 0.3 ml/min, and 300-μl fractions were collected. Bodipy fluorescence in individual fractions was measured on a Victor^3^ multilabel counter (Perkin Elmer) with excitation at 465 nm and emission at 535 nm. Fluorescence that was recovered in fraction (i.e., not retained at top filter of the column) corresponds to disaggregated LPS. Profile calibration with plasma (lipo)proteins was achieved by measuring cholesterol concentration in individual fractions with commercially available enzymatic kit (Cholesterol FS, DiaSys, Holzheim, Germany). Results were expressed as percent of total fluorescence of initial bulk of LPS before size exclusion chromatography.

### Lipopolysaccharides Incubation in Whole Blood

LPS (20 μM) were incubated in a fresh, heparinized pool of whole blood drawn from six wild-type mice. Incubations were performed in the dark for 0–1 h at 37°C with mild agitation and were stopped by transferring reaction tubes on ice at different time points (0, 15, 30, and 60 min). Plasma was separated by a 10-min centrifugation at 8,000 × *g* at 4°C. Blood pellets were washed three times with PBS and split in two aliquots that were constantly kept at 4°C. One aliquot was depleted from erythrocytes with a lysis buffer containing 100 μM EDTA, 150 mM ammonium chloride, and 10 mM potassium bicarbonate and subsequently washed three times with PBS and corresponded to blood leukocytes. Isolated plasmas, total blood pellets, and leukocyte pellets were then submitted to LPS quantitation with the LCMS^2^ method described above. The amounts of LPS associated to erythrocytes were calculated as the difference between LPS in total blood pellets and in corresponding leukocyte pellets. Results were expressed as percent of total bulk of LPS added to blood.

### Lipopolysaccharides Cellular Uptake *in vitro*

Murine hepatocytes (HEPA1-6) or thioglycollate-elicited peritoneal macrophages were plated in six-well plates (5 × 10^5^ cells/well) and cultured respectively in DMEM and RPMI media, both supplemented with penicillin/streptomycin (100 μg/ml each) and 10% total fetal calf serum. Cultures were performed in a water-saturated, 5% CO_2_ atmosphere at 37°C. LPS labeled with DOTA-Bodipy (20 μM) were resuspended in culture media and added to the cells were then cultured for 1 or 24 h. Cells were subsequently detached, and Bodipy fluorescence associated to the cells was quantitated by flow cytometry (LSR II flow cytometer, BD Biosciences) with 488 nm excitation and 530 nm emission filters. LPS uptake was expressed in arbitrary units after correction with initial fluorescence of stock solutions of smooth LPS-Bodipy or rough LPS-Bodipy. Inflammatory response of macrophages was determined by measuring cytokine levels in medium after 24 h by cytometric bead array as described in section “Cytokine Measurements.” All conditions were performed in triplicates. Cell viability at 24 h as evaluated by measuring the ability of cells to adhere to plastic according to the general procedure previously described (Olsson et al., 1982) was similar with both types of LPS treatment, with smooth and rough LPS inducing 36.6 ± 6.2 and 30.4 ± 5.6% mortality after 24 h, respectively (NS, Mann-Whitney test).

### Statistical Analyses

Results are expressed as mean ± S.E. The statistical significance of differences between two data means was determined with the nonparametric Mann-Whitney *U*-test or paired Wilcoxon test as appropriate by using the GraphPad Prism 6 software. *p*’s under 0.05 were considered significant and are indicated with an asterisk.

## Results

### Proinflammatory Potential of Rough Lipopolysaccharides Is Weaker Than That of Smooth Lipopolysaccharides in Mice

**Figure 2:**
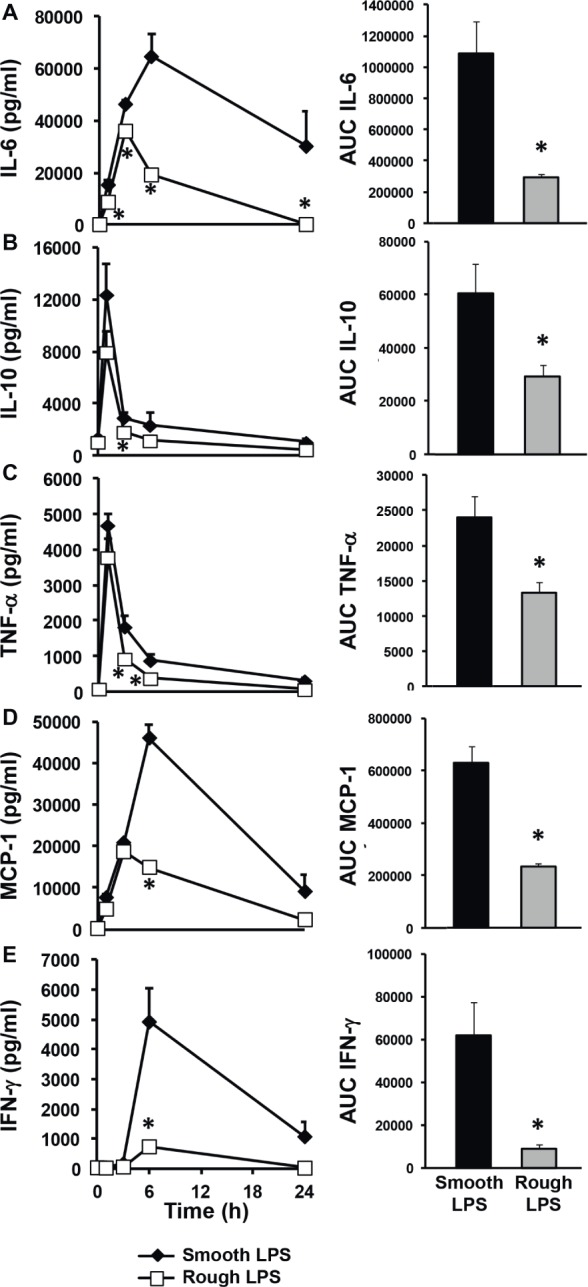
Rough LPS elicits a weaker inflammatory response than smooth LPS *in vivo*. Wild-type C57BL/6 mice were intravenously injected with 200 μl of pyrogen-free saline containing either rough strain LPS (i.e., with a short polysaccharide chain) and smooth strain LPS (i.e., with a long polysaccharide chain) (20 μmol/L each). Blood was collected at the indicated times, and plasma levels of proinflammatory cytokines/chemokines [IL-6 **(A)**, IL-10 **(B)**, TNF-α **(C)**, MCP-1 **(D)**, and IFN-γ **(E)**] were measured by cytometric bead array. Plasma kinetics (left panel) and area under the curve (AUC – right panel) as calculated over the 24-h period are displayed for each cytokine/chemokine in mice injected with smooth LPS (closed diamonds, black bars, *n* = 8) or rough LPS (open squares, gray bars, *n* = 8). In all cases, smooth LPS induced higher cytokine/chemokine production than did rough LPS. Data are expressed as means ± S.E. ^*^Significantly different from smooth LPS, *p* < 0.05. Data are representative of three comparable experiments. See “Experimental Procedures” for additional experimental details.

Identical amounts of smooth (long chain) or rough (short chain) LPS (200 μl at 20 μmol/L) were injected into wild-type mice in order to monitor the kinetics of the inflammatory response *in vivo* over a 24-h period. As shown in Figure 2, both smooth and rough LPS were able to elicit substantial cytokine production with maximal levels observed between 1 and 6 h after injection (Figures 2A–E, left panels). However, for all of the cytokines studied, the kinetic curves obtained with rough LPS were constantly below those obtained with smooth LPS, resulting in significantly lower AUC values over the 24-h period (Figures 2A–E, right panels) (−73, −52, −44, −67, and − 86% for IL-6 (A), IL-10 (B), TNF-α (C), MCP-1 (D), and IFN-γ (E), respectively; *p* < 0.05 in all cases).

### Smooth and Rough Lipopolysaccharides Show Distinct Kinetics in Mouse Plasma and Liver

**Figure 3:**
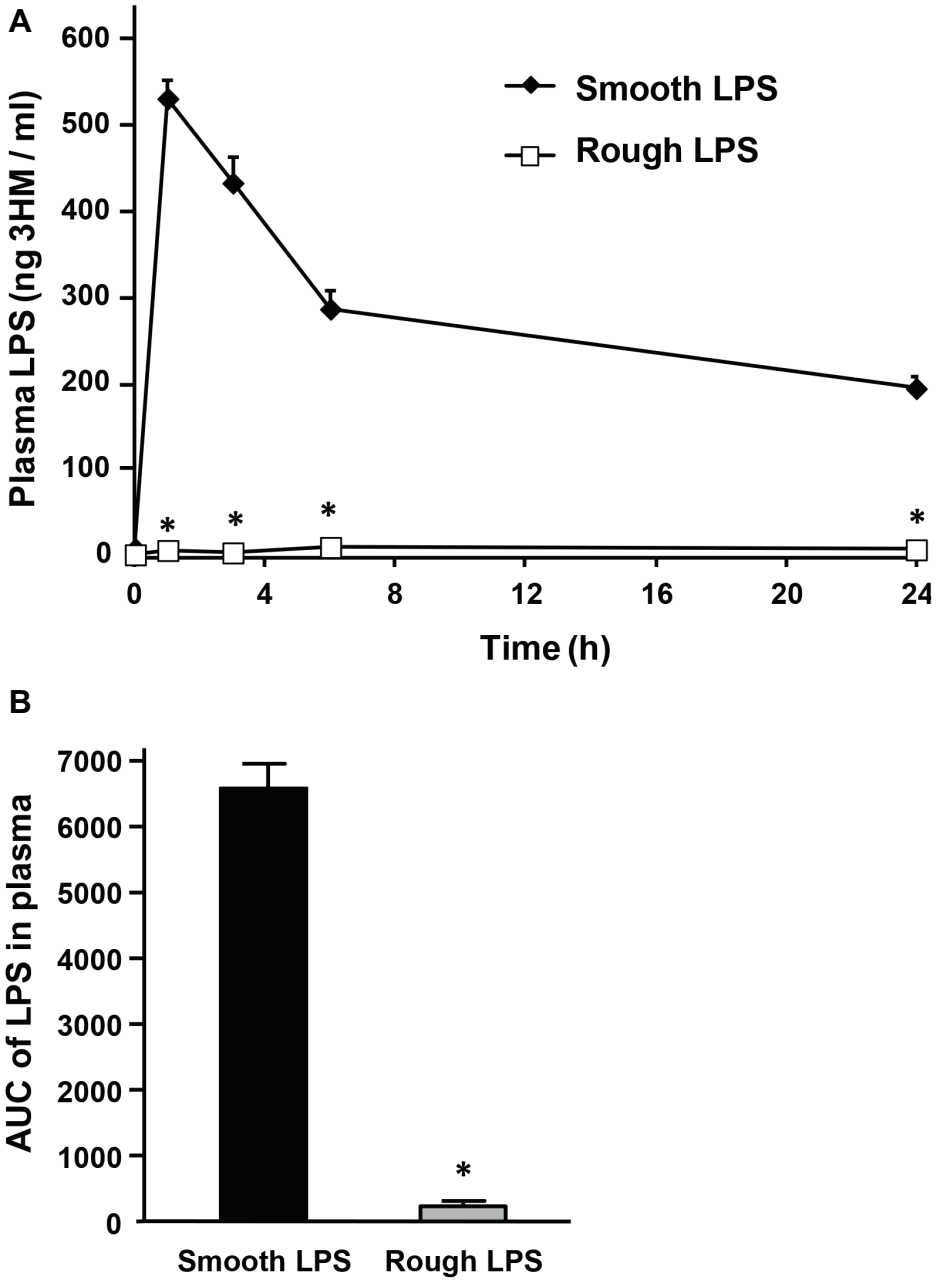
Smooth LPS, unlike rough LPS, has a long residence time in mouse plasma. Wild-type C57BL/6 mice were intravenously injected with 200 μl of pyrogen-free saline containing either smooth or rough LPS (20 μmol/L each). Blood was collected by retro-orbital puncture before injection (time 0) and 1, 3, 6, and 24 h after injection. Plasma levels of smooth and rough LPS were determined by direct quantitation of 3-hydroxymyristate (3HM) as described in “Experimental procedures.” **(A)** Shows plasma kinetics of smooth LPS (closed diamonds, *n* = 8) and rough LPS (open squares, *n* = 8) over a 24-h time period. **(B)** LPS area under the curve (AUC) in total plasma was calculated from kinetic curves measured over 24 h in **A** (black bars, smooth LPS; gray bars, rough LPS). *Significantly different from smooth LPS, *p* < 0.05.

Wild-type mice were intravenously injected with identical amounts of either smooth or rough LPS (200 μl of 20 μmol/L solutions), and the time course of LPS clearance from plasma was assessed by measuring LPS concentration through direct quantitation of LPS mass concentration [3-hydroxy-myristate (3HM) assay] over 24 h. For smooth LPS, and as shown in Figure 3A, a single injection led to high levels of LPS at the 1-h time point. This was followed by a progressive decrease over the period studied. However, substantial amounts (37% of the levels that were detected at the first blood draw after injection) were still present in the circulation at 24 h (Figure 3A, closed diamonds). For rough LPS, and in contrast to smooth LPS, only trace amounts of LPS were measured over the 24-h period (Figure 3A, open squares), with a plasma AUC that was considerably lower for rough LPS than for smooth LPS (Figure 3B, *p* < 0.05).

**Figure 4:**
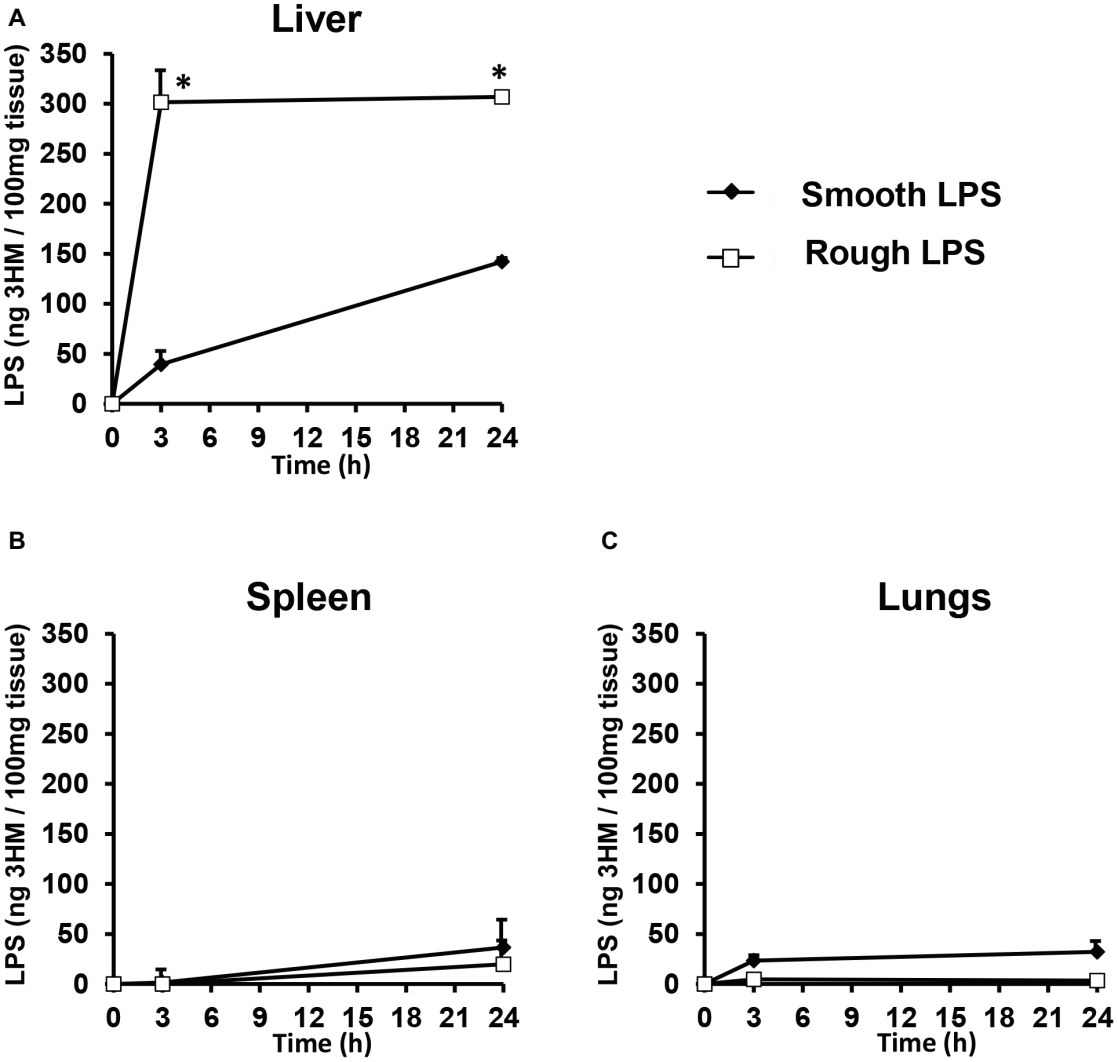
Rough LPS accumulates more rapidly in mouse liver than does smooth LPS. Wild-type C57BL/6 mice were intravenously injected with 200 μl of saline containing either smooth (closed diamonds) or rough (open squares) LPS (20 μmol/L each). Mice were killed by heart puncture 3 or 24 h after injection (*n* = 4 for each group and each time point) and liver **(A)**, spleen **(B)**, and lung **(C)** were collected, immediately frozen in liquid nitrogen and stored at −80°C before further analysis. After homogenization, tissue levels of smooth and rough LPS were determined by direct quantitation of 3-hydroxymyristate (3HM) as described in “Experimental procedures.” Data are shown after subtraction of values obtained from mice injected with 200 μl of pyrogen-free saline and are mean ± SE. ^*^Significantly different from smooth LPS, *p* < 0.05. See “Experimental Procedures” for additional experimental details.

In order to determine the tissue fate of both types of LPS, mice were injected with saline or equal amounts of either smooth or rough LPS. They were killed 3 or 24 h after injection, and LPS levels were measured in the liver, spleen, and lungs. As shown in Figure 4, the majority of LPS accumulated in the liver (Figure 4A); very low amounts of LPS were recovered in the spleen (Figure 4B) and in the lungs (Figure 4C). High amounts of rough LPS accumulated in the liver as early as 3 h after injection. In contrast, smooth LPS was found at low levels in this organ at the same time point, with values that were 87% lower than for rough LPS (*p* < 0.05; Figure 4A). Twenty-four hours after injection, rough LPS levels in the liver were higher than smooth LPS levels (−54%, *p* < 0.05; Figure 4A).

### Smooth and Rough Lipopolysaccharides Form Aggregates of Different Shapes

**Figure 5:**
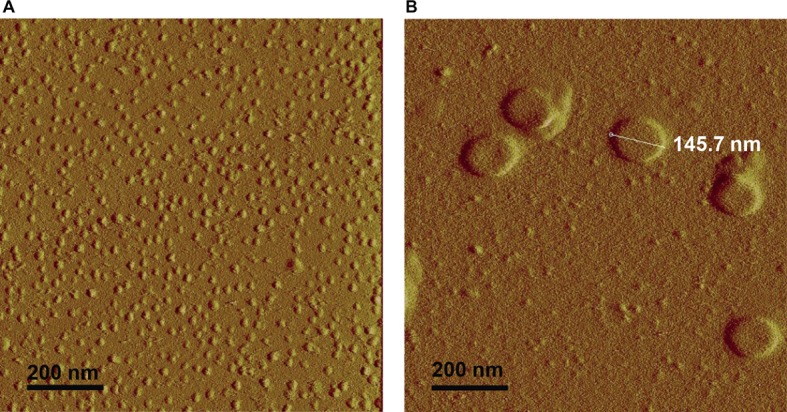
Atomic force microscopy shows that rough LPS forms larger aggregates than smooth LPS. Saline solutions containing 20 μmol/L of smooth **(A)** or rough LPS **(B)** were analyzed with an atomic force microscope (see “Experimental Procedures” for additional experimental details). **(A)** is a representative view of small-sized aggregates of smooth LPS. **(B)** is a representative view of large-sized aggregates of rough LPS.

Smooth and rough LPS were dissolved in saline at identical concentrations (20 μmol/L) and were first analyzed by atomic force microscopy (Figure 5). Both LPS solutions contained aggregates. They were apparently homogeneous in each preparation but differed markedly from one LPS type to the other. Smooth LPS formed small, simple globular aggregates with an apparent diameter ranging from 20 to 30 nm (Figure 5A). Rough LPS formed larger aggregates (apparent diameter, 100–200 nm) with simple to multilobular spherical shapes (Figure 5B).

**Figure 6:**
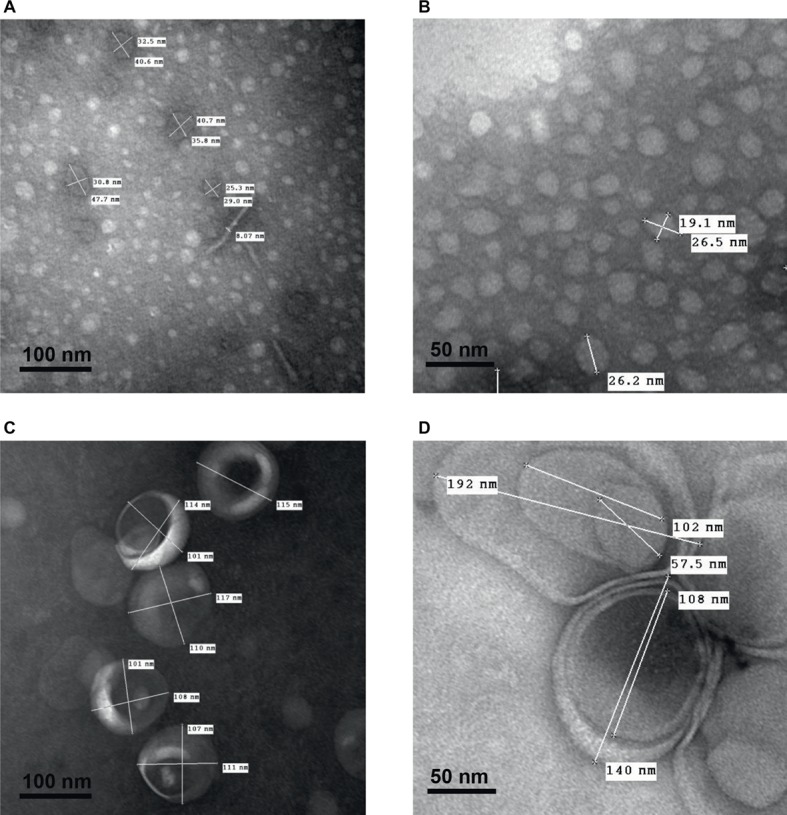
Transmission electron microscopy after negative staining shows multilamellar vesicles formed by rough LPS and small aggregates formed by smooth LPS. Saline solutions containing smooth LPS (**A,B**, 15 μmol/L) or rough LPS (**C**, 22 μmol/L and **D**, 15 μmol/L) were analyzed with a transmission electron microscope after staining with phosphotungstic acid (**A,C**; black bars, 100 nm) or ammonium molybdate (**B,D**; black bars, 50 nm) (see “Experimental Procedures” for additional experimental details). Pictures are representative of several independent experiments.

As shown in Figure 6, transmission electron microscopy observations of smooth and rough LPS solutions (concentration ranging from 15 to 22 μmol/L) after negative staining confirmed the different sizes and shapes of the aggregates formed by the two compounds. Consistent results were obtained with phosphotungstic acid (Figures 6A,C) and ammonium molybdate (Figures 6B,D) staining techniques. In addition, both staining techniques revealed that smooth LPS aggregates formed homogeneous, dense micelle-like structures (Figures 6A,B), while rough LPS appeared to form diffuse unilamellar or multilamellar vesicles (Figures 6C,D).

### Smooth and Rough Lipopolysaccharides Form Aggregates With Different Physical Properties and Stability

Smooth and rough LPS were solubilized in saline at a final concentration of 20 μmol/L (similar to the concentration used for atomic force microscopy) in order to get further insights into the physical properties of the aggregates formed with the two compounds. The parameters studied were hydrodynamic size, hydrophobicity, and zeta potential.

**Figure 7:**
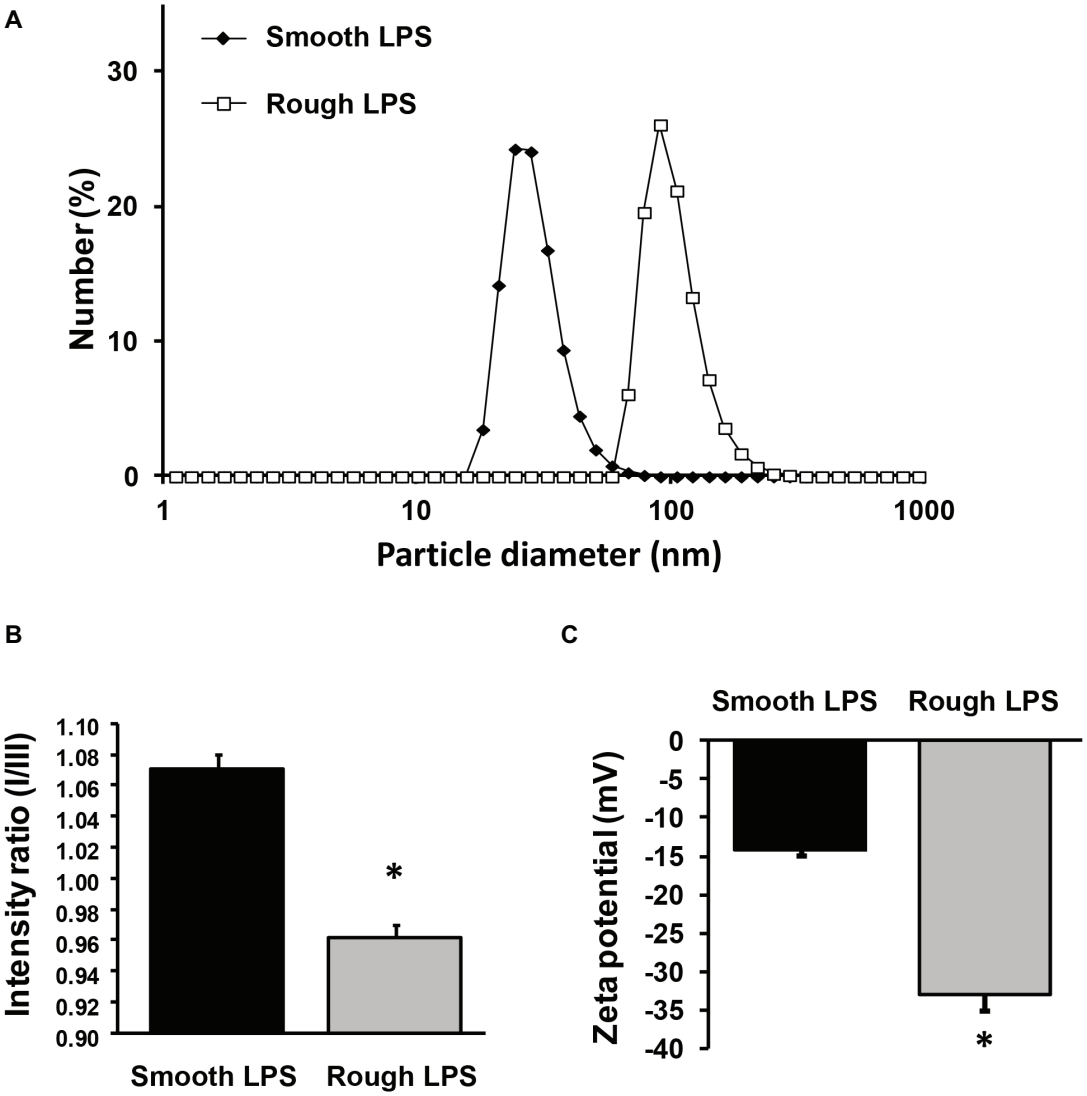
Size distribution, zeta potential, and hydrophobicity index of structures formed by smooth and rough LPS. Size distribution of smooth LPS (closed diamonds) and rough LPS (open squares) in saline was determined by dynamic light scattering **(A)**. Hydrophobicity index **(B)** and zeta potential **(C)** of smooth (black bars) and rough LPS (gray bars) was determined by spectrofluorimetry using a pyrene probe and by zetametry, respectively. See “Experimental Procedures” for additional experimental details. Bars are mean ± SE of quadruplicate measurements. ^*^Significantly different from smooth LPS, *p* < 0.05. All panels are representative of three distinct experiments.

Hydrodynamic size was determined by dynamic light scattering (Figure 7A). In solution, both smooth and rough LPS produced single peaks with near-Gaussian distribution, indicating that each compound formed a mostly homogeneous aggregate population. In good agreement with microscopy analyses (Figures 5, 6), smooth LPS aggregates in solution were clearly smaller than rough LPS aggregates, with mean hydrodynamic peaks at 24.4 and 91.3 nm, respectively (Figure 7A).

Aggregate hydrophobicity was assessed by steady-state fluorescence using a nonpolar pyrene probe. As shown in Figure 7B, the ratio of peak I to peak III fluorescence intensities was significantly lower for rough LPS than for smooth LPS (0.963 ± 0.007 *versus* 1.070 ± 0.010; respectively; *p* < 0.05). This indicates that the microenvironment within the aggregates is more hydrophobic with rough LPS than with smooth LPS.

Zeta potential values, which represent the electric charges at the periphery of the aggregates in suspension, were measured by electrophoretic light scattering. As shown in Figure 7C, smooth and rough LPS-derived aggregates gave values of −14.2 ± 0.9 and −33.0 ± 2.1 mV, respectively (*p* < 0.05). This indicates that repulsion forces of aggregates containing rough LPS are greater than those of aggregates containing smooth LPS.

**Figure 8:**
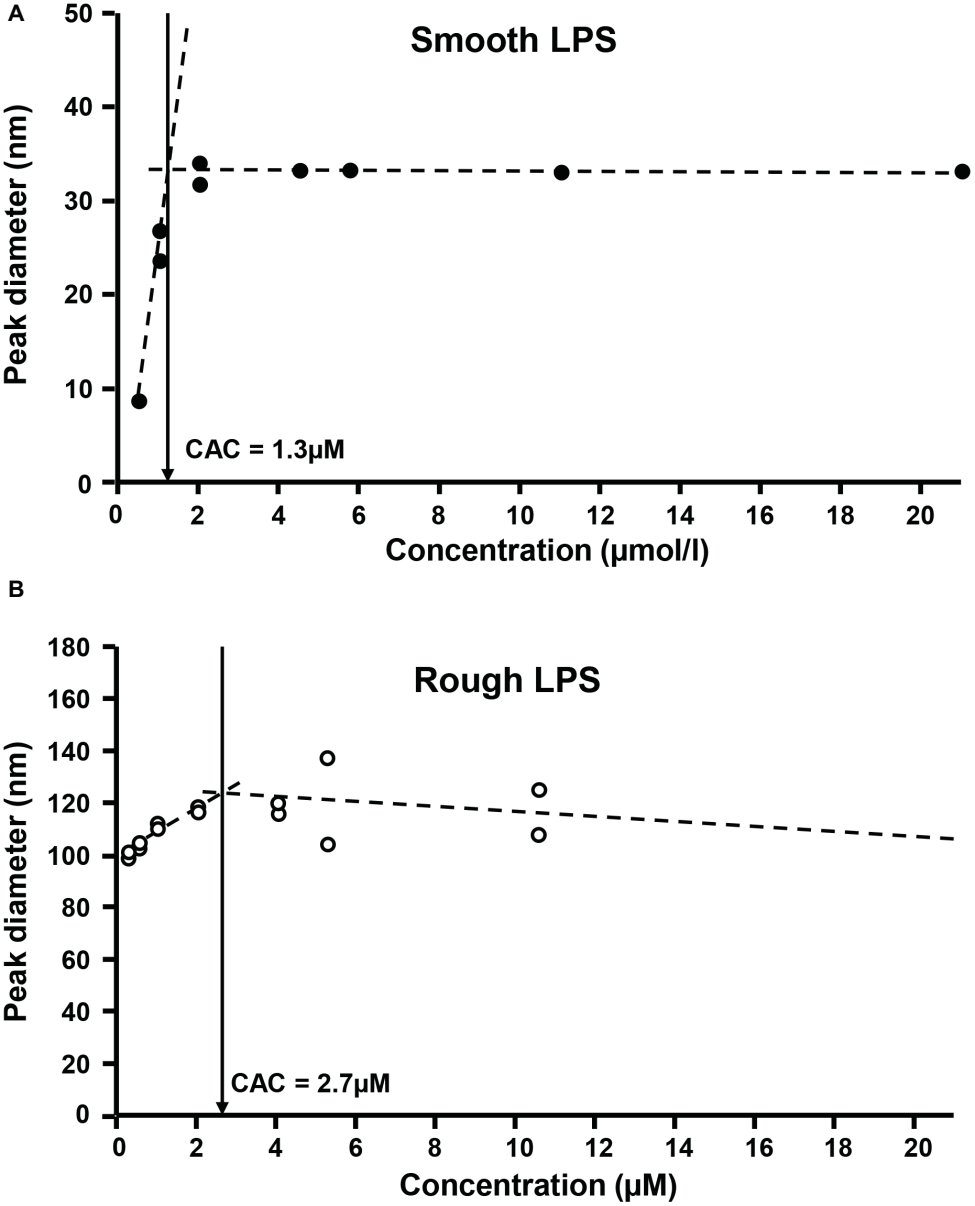
Rough LPS has a higher critical aggregation concentration than smooth LPS. Critical aggregation concentration (CAC) of smooth LPS **(A)** and rough LPS **(B)** in saline was calculated after plotting aggregate size (as determined by dynamic light scattering) against LPS concentration. For both LPS, aggregation profiles were distributed along two linear regression curves (dotted lines). The CAC corresponds to the intersection (arrows) of the two linear curves, below which aggregates start to decrease in size. See “Experimental Procedures” for additional experimental details.

Finally, critical aggregation concentrations (CAC) of the two LPS types were determined as an additional indicator of aggregability. The formation of aggregates with smooth and rough LPS was monitored by dynamic light scattering, using increasing concentrations in a saline solution. As shown in Figure 8, aggregates of smooth LPS (Figure 8A) appeared to be more stable than aggregates of rough LPS (Figure 8B), with CAC values of 1.3 and 2.7 μmol/L, respectively.

### Smooth and Rough Lipopolysaccharides Show Different Abilities to Disaggregate in Plasma and to Associate to Lipoproteins, Blood Cells, and Liver Cells

**Figure 9:**
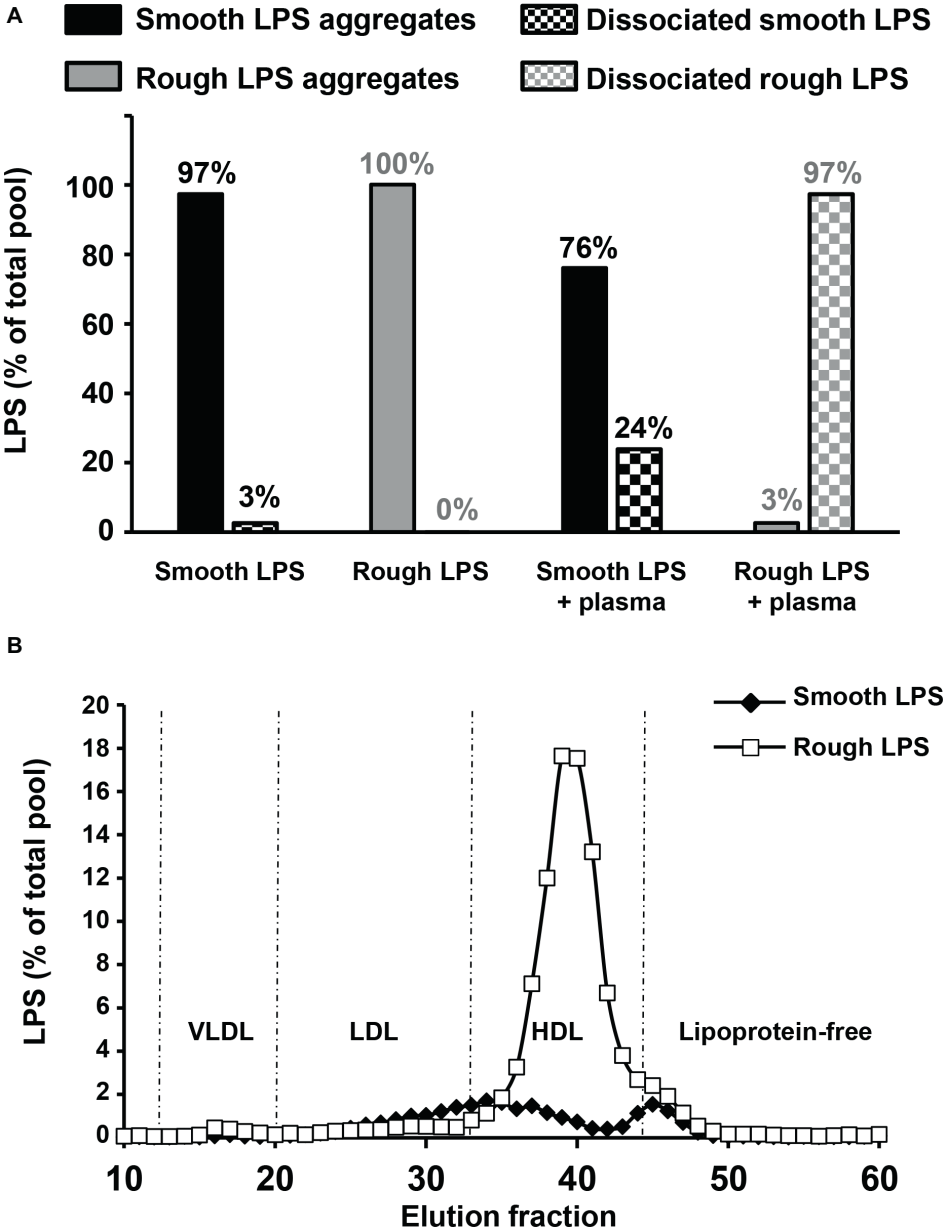
Rough LPS aggregates dissociates more readily than smooth LPS in plasma. Smooth and rough LPS (20 μM each) were labeled with Bodipy and incubated for 20 min at 37°C in the presence of saline plasma isolated from wild-type mice and were subsequently subjected to size exclusion chromatography. Recovery and distribution of LPS was determined by fluorescence reading. See “Experimental Procedures” for additional experimental details. **(A)** shows the ratios of smooth (black bars) and rough LPS (gray bars) that were recovered as large aggregates (full bars) or dissociated molecules (checked bars) after incubation in saline (left) or in plasma (right). Values above each bar indicate the amount of each LPS form as percent of total pool. **(B)** shows plasma distribution of the dissociated forms of smooth (closed diamonds) or rough LPS (open squares).

In order to test the effect of plasma on LPS aggregate stability, smooth ad rough LPS aggregates were incubated in the presence of saline of plasma before separation of remaining aggregates and LPS that associated with plasma components by size exclusion chromatography. As shown in Figure 9A, in the absence of plasma, both smooth and rough LPS expectedly formed large-sized aggregates that were retained at the top of the chromatography column (aggregates, 97% for smooth LPS; 100% for rough LPS; Figure 9A, left). The presence of plasma induced the disaggregation of only 24% of smooth LPS, while it led to the disaggregation of the totality (97%) of the bulk of rough LPS (Figure 9A, right). Disaggregated LPS showed different distribution patterns in plasma. (Figure 9B) with smooth LPS associated to LDL (low-density lipoproteins), HDL (high-density lipoproteins), and plasma proteins in similar proportions, while rough LPS mainly bound to plasma HDL.

**Figure 10:**
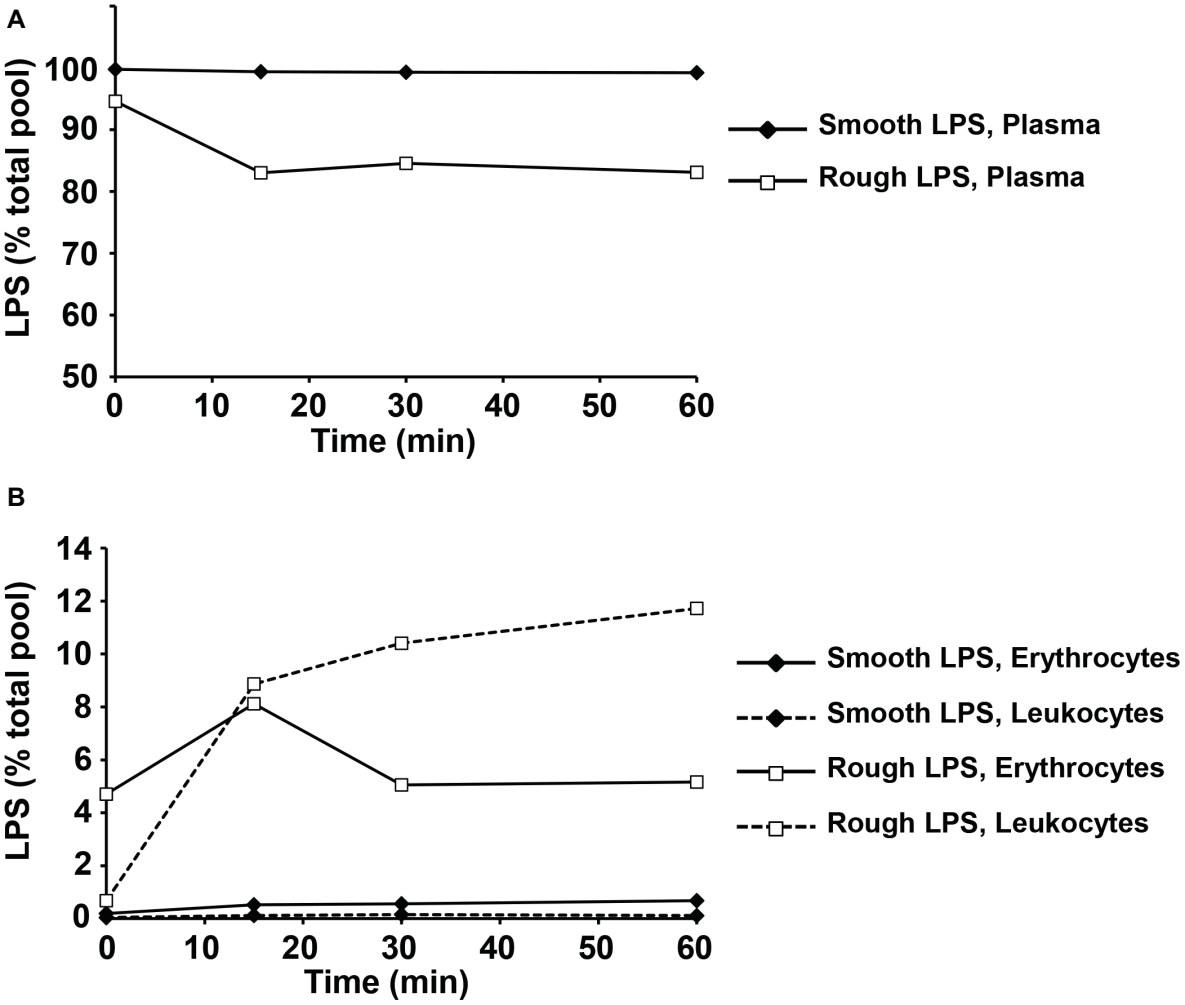
Rough LPS is transferred more readily than smooth LPS from plasma circulating cells in whole blood *ex vivo*. Smooth LPS (closed diamonds) or rough LPS (open squares; 20 μM each) were incubated in whole blood from wild-type mice for 0, 15, 30, or 60 min at 37°C prior to separation of plasma **(A)**, erythrocytes and leukocytes **(B)** and direct quantitation of 3-hydroxymyristate (3HM) as described in “Experimental procedures.” Results are expressed as percent of total pool added to whole blood.

**Figure 11:**
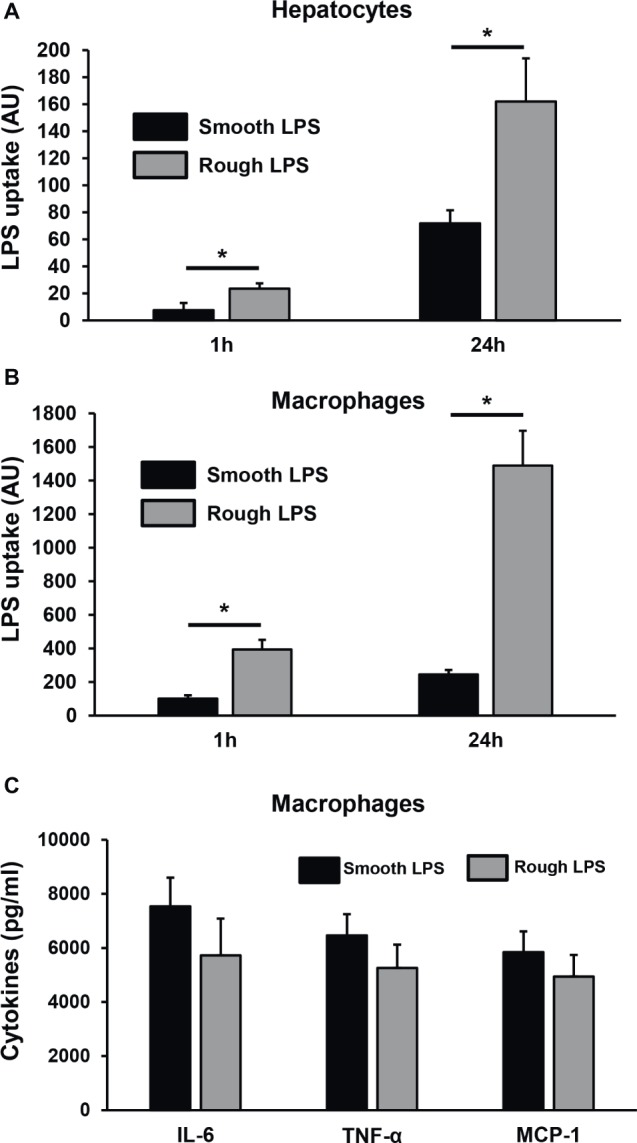
Rough LPS associates more readily than smooth LPS to hepatocytes and macrophages in culture. Smooth LPS (black bars) and rough LPS (gray bars; 20 μM each) were labeled with Bodipy and incubated with murine hepatocytes (HEPA1–6; **A**) or thioglycollate-elicited macrophages **(B,C)** in culture media supplemented with fetal calf serum for 1 or 24 h. Cells were subsequently detached, Bodipy fluorescence associated to the cells was quantitated by flow cytometry, and LPS uptake was expressed in arbitrary units after correction with initial fluorescence of stock solutions of smooth LPS-Bodipy or rough LPS-Bodipy. **(C)** shows inflammatory cytokine levels in culture media from the peritoneal macrophages after hours incubation. See “Experimental Procedures” for additional experimental details. Bars are mean ± SE of quadruplicate measurements. ^*^Significantly different from smooth LPS, *p* < 0.05.

Next, smooth and rough LPS were incubated *ex vivo* in the presence of fresh, total blood from wild-type mice before quantification of LPS remaining in plasma (Figure 10A) or associated to erythrocytes or leukocytes (Figure 10B). While the totality of smooth LPS remained in plasma, with no association to blood cells over 1 h of incubation, 16.5% of rough LPS left the plasma compartment after 15 min and associated to blood cells, with a majority of rough LPS bound to leukocytes after 1 h of incubation. Finally, when incubated in the presence of murine hepatocyte cell line or peritoneal macrophages, rough LPS once again showed larger uptake by cultured cells at both early (1 h) and late (24 h) time points, with, respectively, 4.6 and 2.5 times increases in hepatocytes (Figure 11A) and 3.1 and 2.3 increases in macrophages (Figure 11B) compared with smooth LPS (*p* < 0.05 in all cases). Strikingly, the larger uptake of rough LPS by macrophages was not associated with increased inflammatory response when compared with macrophages treated with smooth LPS (Figure 11C).

## Discussion

In the present study, it is shown that smooth LPS has a significantly higher proinflammatory potency than rough LPS when used at identical molar concentrations *in vivo*. The marked differences in biological effects of rough and smooth LPS were most likely due to their distinct physicochemical and pharmacokinetic features. Indeed, whereas smooth LPS produced a substantial initial peak in mouse plasma followed by a slow decay over the 24-h period, rough LPS was barely detectable over the period studied. This suggests that rough LPS is rapidly cleared, as confirmed by its concomitant and rapid accumulation in the liver as early as 3 h after injection. This finding brings new support to a reverse lipopolysaccharide transport pathway (RLT) that today has been deciphered and imaged (Mathison and Ulevitch, 1979; Freudenberg et al., 1980, 1982; Harris et al., 1993; Cai et al., 2008; Gautier and Lagrost, 2011; Lu and Munford, 2011; Duheron et al., 2014). This pathway requires four different steps: (1) dissociation of LPS aggregates, with the shedding of single LPS molecules, (2) transfer of single LPS molecules to circulating lipoproteins, (3) liver uptake lipoprotein-borne LPS, and (4) biliary secretion of LPS processed by the liver. This general scheme puts the liver as the primary organ for LPS uptake and disposal (Mathison and Ulevitch, 1979; Freudenberg et al., 1982). Although the precise modalities of plasma LPS transport in our *in vivo* model would require further investigations, our hypothesis is that after intravenous injection, circulating smooth LPS, which form more stable aggregates, undergo the first step of RLT in a very slow fashion, resulting in accumulation in plasma at the expense of liver uptake (step 3 of RLT). On the opposite, circulating rough LPS are quickly disaggregated (very short residence time in plasma) and taken up by the liver, leading to immediate hepatic accumulation. The more rapid clearance of rough LPS from the circulation by the liver, as reported in the present study, is a cogent explanation to the reduced inflammatory response it elicited as compared with smooth LPS.

It should be emphasized that in our case the LPS preparations were injected into mice as aggregates since the preparations were at a concentration of 20 μmol/L, clearly above the CAC value [present study and Aurell and Wistrom (1998)]. This indicates that the differences observed most likely relate to the structural features and/or pharmacokinetics of the aggregates. In support of this view, physicochemical analyses showed that aggregates of smooth and rough LPS displayed markedly different structural features as illustrated by distinct size. Indeed, and as observed by electron transmission microscopy and atomic force microscopy, aggregates of smooth LPS were 5–10 times smaller than aggregates of rough LPS. These observations were confirmed by DLS analysis of aggregates in saline solution, with a mean size of 24.4 and 91.3 nm for smooth and rough LPS, respectively. These values are consistent with those reported by others, with aggregates of smooth LPS in the 10–50 nm diameter range (Bergstrand et al., 2006; Hardy et al., 2012) and aggregates of rough LPS in the 50–100 nm diameter range (Santos et al., 2003; Sasaki and White, 2008; D’Errico et al., 2010), depending on the technique used (Brogden and Phillips, 1988; Aurell and Wistrom, 1998). Of note, our study compared the aggregate size of LPS molecules from the same bacterial species and original serotype with identical lipid A and inner core moieties but differing in the polysaccharide chain length only. Consequently, it brings new support to the polysaccharide chain length as a major determinant of the structure of LPS aggregates.

In this context, it must be stressed that circulating aggregates or bacterial blebs, rather than free LPS monomers, have been recognized as the active form of LPS in aqueous media (Mueller et al., 2004, 2005). Since LPS molecules have been reported to interact with CD14, TLR4, and MD2 mainly as monomers (Tobias et al., 1995; Park et al., 2009), this implies that activation of the innate immune response triggered by initial binding to the receptor complex requires the release of LPS monomers from either circulating aggregates (Gioannini et al., 2004) or aggregates bound to the immune cell surface (Mueller et al., 2005). This indicates that various structural features might actually modulate the stability, pharmacokinetic characteristics, and proinflammatory activity of LPS aggregates. In earlier studies, no clear picture could be provided, and the greater inflammatory potential was alternatively attributed to large (Mueller et al., 2004, 2005) or small (Vukajlovich and Morrison, 1983) aggregates. Others reported no relationship between aggregate size and inflammatory potential (Kitchens and Munford, 1998; Hardy et al., 2012). The present study sheds new light on the intrinsic nature of LPS aggregates, here with rough LPS forming multilobular/multilamellar structures and with smooth LPS forming simple, spheroidal aggregates (Figures 5, 6). These structural features most likely reflect differences in LPS behavior in an aqueous environment, given that the amphipathic nature of these molecules determines their ability to form aggregates in solutions (Sasaki and White, 2008). Because smooth LPS contain a large hydrophilic moiety, it was able in the present study to produce aggregates with a low hydrophobicity index and with high stability in the aqueous phase. Accordingly, we found a CAC value for smooth LPS that was half that for rough LPS (Figure 8). This view regarding CAC adds to earlier studies, which showed that LPS species with a long polysaccharide chain have a CMC/CAC value around 1 μmol/L, while LPS with a short polysaccharide chain have a CMC/CAC value closer to 5 μmol/L (Aurell and Wistrom, 1998; Santos et al., 2003; Bergstrand et al., 2006). The hypothesis that rough LPS forms less stable aggregates is further sustained in the present study by zeta potential data reflecting the repulsion forces (Lyklema, 1991; Domingues et al., 2012; Hardy et al., 2012; Singh et al., 2013).

Finally, we could directly demonstrate that rough LPS aggregates are less stable in *ex vivo* experiments mimicking the patho-physiological situation of endotoxemia, i.e., when incubated in the presence of either plasma or whole blood. We could show that less than 25% of smooth LPS could undergo disaggregation and associate to plasma components, while almost 100% of rough LPS could disaggregate in the same experimental setup after only 20 min of incubation in the presence of plasma only. It is worth to note that unlike disaggregated smooth LPS, rough LPS monomers were mainly bound to plasma HDL which, due to their well-recognized role as LPS scavengers in the circulation (Munford et al., 1981; Cavaillon et al., 1990), could contribute to their quick clearance from the plasma. Accordingly, when incubated in the presence of whole blood, substantial amounts of rough LPS were transferred from the plasma compartment to circulating cells within 15 min, while the whole bulk of rough LPS almost exclusively remained in plasma over a 1-h incubation. However, transfer of rough LPS to the circulating cells could not exceed 16% of the total bulk of LPS, indicating that the maximal acceptor capacities of blood cells are quickly reached in our model of isolated blood. In the presence of hepatocytes or macrophages which were cultured in media supplemented with complete serum that contains lipoproteins, the magnitude of accumulation of rough LPS was once again much higher (2.3–4.6 times) than that of smooth LPS, but this time LPS cellular uptake did not saturate after 1 h and could rise until 24 h of incubation. This indicates that hepatocytes and macrophages/Kupffer cells, i.e., two cell types involved in LPS clearance in the liver (Hampton et al., 1991; Shao et al., 2012), are much more active in the uptake of plasma-derived LPS than circulating cells. In addition, the fact that the increased uptake of rough LPS does not translate into increased inflammatory response of treated macrophages suggests that the cellular components involved in additional accumulation of rough LPS differ from the receptors involved in the triggering of the inflammatory cascade. Whether this specific effect might relate to the expression of receptors such as SR-BI, LDLR, or CD36, which could be involved in lipoprotein-borne LPS monomers, will deserve further studies. In parallel, these latter results also indicate that, in a closed system, equimolar amounts of rough and smooth LPS with identical lipid A moiety display equivalent intrinsic inflammatory potency on the long term. Taken together, our *ex vivo* experiments suggest that polysaccharide chain length is a rate-limiting factor of the first step of the RLT pathway which involves (1) LPS disaggregation, (2) transfer of LPS monomers to lipoproteins, and (3) clearance of lipoprotein-borne LPS by the hepatobiliary tract. Interestingly, it was shown in the *Salmonella* genus that LPS with long polysaccharide chains, when compared with LPS from rough strains, can enhance the virulence of bacteria by slowing down macrophage-mediated antigen processing and presentation to T cells, probably through masking specific bacterial epitopes (Zirk et al., 1999). The relative contribution of this mechanism compared to the effect of polysaccharide chain on LPS aggregate stability and RLT described in the present work as well as the possible synergy of both phenomena to the deleterious effects of bacterial infection would deserve further attention.

Overall, the present study highlights that the length of the polysaccharide chain of LPS is a major determinant of the nature and stability of the aggregates it forms in aqueous and biological media. Polysaccharide chain length is shown to have a significant impact on the pharmacokinetics, clearance and thus the inflammatory properties of LPS when injected as purified preparations *in vivo*. Here, two possible explanations may account for the more rapid clearance of rough LPS aggregates from plasma. First, large aggregates of rough LPS allow the docking and internalization of a higher number of LPS molecules per particle than is the case for small aggregates (Mathison and Ulevitch, 1979; Kitchens et al., 1998; Kitchens and Munford, 1998). Second, large aggregates of rough LPS are less stable than small aggregates of smooth LPS in aqueous media and are thus more rapidly disaggregated and cleared from the circulation (Gautier et al., 2008, 2010). Regarding this latter point, our experiments clearly showed that aggregate stability and propensity of LPS to dissociate as monomers to interact with plasma lipoproteins in a first step and with cells in a second step are largely determined by the length of polysaccharide chain length. Because the injection of LPS preparations is commonly used to trigger inflammation in the research laboratory, polysaccharide chain length, zeta potential, and overall aggregability of LPS should be taken into account to predict the proinflammatory effect that can be expected in experimental settings. In addition, better knowledge and control of LPS aggregation and disaggregation might lead to new strategies to enhance the RLT pathway.

## Data Availability

The raw data supporting the conclusions of this manuscript will be made available by the authors, without undue reservation, to any qualified researcher.

## Ethics Statement

All experiments were performed in accordance with institutional guidelines and approved by the Ethics Committee of the University of Burgundy (Protocol number 2511).

## Author Contributions

WS performed animal kinetics experiments, physical characterization of LPS aggregates and wrote the manuscript. J-PB performed LPS quantitation by LCMS^2^. JL, VaD, NLG, and TG performed animal experiments. ViD set up the fluorescent labeling technique for LPS. DB, DC, EL, and BG performed physical characterization of LPS aggregates. MM and FD produced the fluorescent label for LPS. WS, LL, and TG designed the experiments and analyzed experimental results. CP and DP performed cell culture experiments. DM performed manuscript editing. TG performed aggregation stability experiments and wrote the manuscript.

### Conflict of Interest Statement

The authors declare that the research was conducted in the absence of any commercial or financial relationships that could be construed as a potential conflict of interest.

## Abbreviations

CAC: Critical aggregation concentration
CD14: Cluster of differentiation 14
CMC: Critical micellar concentration
DLS: Dynamic light scattering
3HM: 3-Hydroxymyristate
IFNγ: Interferon gamma
IL: Interleukin
LCMS^2^: Liquid chromatography/tandem mass spectrometry
LPS: Lipopolysaccharide(s)
MCP-1: Macrophage chemoattractant protein 1
MD2: Myeloid differentiation factor 2
TLR: Toll-like receptor
TNFα: Tumor necrosis factor alpha.
